# Modification of the Two-Point Scaling Theory for the Description of the Phase Transition in Solution. Analysis of Sodium Octanoate Aqueous Solutions

**DOI:** 10.1007/s10953-012-9795-6

**Published:** 2012-02-02

**Authors:** Henryk Piekarski, Michał Wasiak, Leszek Wojtczak

**Affiliations:** 1 Department of Physical Chemistry, University of Łódź, Pomorska 165, 90-236 Łódź, Poland; 2 Department of Solid State Physics, University of Łódź, Pomorska 149/153, 90-236 Łódź, Poland

**Keywords:** Scaling theory, Phase transition, Colloids, Heat capacity, Volume

## Abstract

On the basis of conventional scaling theory, the two-point scaling theory was modified in order to describe the influence of composition on the partial molar heat capacity and volume during the micellization process. To verify the theory, isobaric heat capacities and densities of aqueous sodium octanoate solutions were measured over wide composition and temperature ranges and the modified approach was used to analyze the calculated partial molar heat capacities and volumes of the surfactant in water. The results obtained indicate that the micellization process is subject to the scaling laws. The results were compared with those for other systems. Peculiar behavior of the critical indices was observed and correlated with the structure of the micelles.

**Electronic Supplementary Material:**

The online version of this article (doi:10.1007/s10953-012-9795-6) contains supplementary material, which is available to authorized users.

## Introduction

Research on colloidal systems is carried out using many different experimental techniques. Interpreting the experimental data requires a model which combines a description of the thermodynamics together with a description of the structure. One of such models is scaling theory [1], particularly the two-point scaling theory introduced by Wojtczak et al. [2, 3] for the description of paramagnetic–ferromagnetic phase transitions, which extends the theory to the case of noncontinuous phase transitions. This idea came from the fact that some analogies can be perceived between the phase transitions that occur in the two systems (i.e. paramagnetic–ferromagnetic and regular solution–micellar systems): a dominant role is played by surface effects and changes in degree of order of surface particles. Moreover, the similarity between the shape of the curve describing the partial molar heat capacities at constant pressure of surfactant on molality, and the shape of the temperature dependence of the specific heat capacities under the constant external field in the case of magnetic systems, gave us additional encouragement to consider whether phase transitions in these solutions also follow scaling laws.

Our research showed indeed that the thermodynamic relations following from the scaling theory can be used for the description of colloidal [4] and microheterogeneous systems [5, 6]. The application of the theory, however, was completely “intuitive” and, in order to interpret the results, a reformulation of the theory with respect to the original basis was necessary.

## Theoretical Study

In physics, critical phenomena is a term related with critical points, which are conditions at which a phase boundary ceases to exist. For chemists, commonly known critical points are the critical temperature (or pressure) at which vapor pressure curve terminates, or in liquid systems the critical temperature of mixing. Another kind of critical phenomena are the paramagnetic–ferromagnetic phase transitions, which are described by the conventional scaling theory [1].

Scaling theory assumes that thermodynamic potentials are homogeneous functions with respect to the external field and the reduced temperature *ε*. According to Stanley [1] the static scaling hypothesis states that they are of more general form, namely: *f*(*λ* ^*a*^ *x*,*λ* ^*b*^ *y*)=*λf*(*x*,*y*) rather then *f*(*λx*,*λy*)=*λ* ^*p*^ *f*(*x*,*y*). The reduced temperature is defined as:

1 $$\varepsilon =\frac{|T-T_{c}|}{T_c} $$

where *T*_*c*_ is the critical temperature.

The external field term can have many meanings. In the conventional theory it was the magnetic field [1] but it can be the pressure *p* when solutions are considered.

Application of scaling theory to the description of solutions requires the introduction of one more variable, namely the reduced amount of solute *μ* defined as:

2 $$ \mu= \frac{\vert n_{2} - n_{c} \vert}{n_{c}}$$

where *n*_2_ is the amount of the solute and *n*_*c*_ is the critical amount of the solute; by analogy to the conventional theory it is the amount of solute at which the critical phenomenon occurs.

As a result the thermodynamic potential, in our case the Gibbs energy, can be expressed as a function of three variables:

3 $$G = G(\varepsilon,p,\mu) $$

When the amount of solvent *n*_1_ is constant and its mass is equal to 1000 g, the amount of solute *n*_2_ is equal to the molality of solution by means of:

4 $$ n_{1} = \frac{1000}{M_{1}} \quad \Rightarrow \quad n_{2} = m$$

which allows use of the molality instead of the amount of solute, and equates the critical amount of the solute with the critical micellar concentration *c.m.c.*

The conventional theory describes the behavior of the molar properties of pure substances. In the case of solutions, partial molar quantities should be considered instead. The singularities in the behavior of thermodynamic functions related to the critical phenomena in solutions occur with increasing molality, not temperature, as in the case of conventional theory.

On the basis of the fundamental assumption of scaling theory about the homogeneity of thermodynamic potentials, we can write:

5 $$ \lambda G(\varepsilon,p,\mu) = G\bigl(\lambda^{a}\varepsilon,\lambda^{b}p,\lambda^{c}\mu\bigr)$$

for any *λ* and arbitrary *a*, *b*, *c* (non-vanishing simultaneously).

Differentiating both sides of Eq. 5 we obtain:

6 $$ \lambda\frac{\partial^{2}G(\varepsilon,p,\mu )}{\partial p\partial n} = \frac{\partial^{2}G(\lambda^{a}\varepsilon ,\lambda^{b}p,\lambda^{c}\mu)}{\partial p\partial n} =\pm\frac{1}{n_{c}} \lambda^{b}\lambda^{c}\frac{\partial^2G(\lambda^{a}\varepsilon,\lambda^{b}p,\lambda^{c}\mu )}{\partial ( \lambda^{b}p )\partial ( \lambda^{c}\mu )}$$

Taking into account the thermodynamic fundamentals $(\frac{\partial^{2}G}{\partial p\partial n_{2}} )_{T,n_{1}} = V_{2}$ and applying it to the homogeneous function:

7 $$\biggl( \frac{\partial^{2}G(\lambda^{a}\varepsilon ,\lambda^{b}p,\lambda^{c}\mu)}{\partial( \lambda^{b}p )\partial(\lambda^{c}\mu)} \biggr)_{T,n_{1}} = V_{2}\bigl(\lambda^{a}\varepsilon,\lambda^{b}p,\lambda^{c}\mu\bigr) $$

we obtain:

8 $$\pm\frac{1}{n_{c}}\lambda V_{2}(\varepsilon,p,\mu) = \pm \frac{1}{n_{c}}\lambda^{b +c}V_{2}\bigl(\lambda^{a}\varepsilon,\lambda^{b}p,\lambda^{c}\mu\bigr) $$

and:

9 $$V_{2} = \lambda^{b + c -1}V_{2}\bigl(\lambda^{a}\varepsilon,\lambda^{b}p,\lambda^{c}\mu \bigr) $$

Since Eq. 9 is valid for all values of *λ* it must hold also for the particular choice:

10 $$\lambda= \mu^{ - \tfrac{1}{c}} $$

which is equivalent to:

11 $$\lambda^{c}\mu= 1 $$

If we consider a constant (or zero, in conventional theory) field (the pressure in our case), constant temperature, and the above (Eqs. 10 and 11) value for *λ*, then the expression *V*_2_(*λ* ^*a*^ *ε*,*λ* ^*b*^ *p*,*λ* ^*c*^ *μ*) becomes constant and we denote it as $V_{2}^{\mathrm{o}}$. This leads to:

12 $$ V_{2} = \mu^{\tfrac{1 - b - c}{c}}V_{2}^{\mathrm{o}}$$

Taking into account the above results, the equation for the partial molar volume, as a function of solution molality, at constant pressure and temperature, can be derived in the form of a power law as follows:

13 $$ V_{2} = V_{2}^{\mathrm{o}}\mu^{\beta'}$$

with the critical index *β*′:

14 $$ \beta' = \frac{1 - b - c}{c}$$

A similar relation can be derived for the partial molar heat capacity:

15 $$ \biggl( \frac{\partial^{3}G(\lambda^{a}\varepsilon ,\lambda^{b}p,\lambda^{c}\mu)}{\partial ( \lambda^{a}\varepsilon )^{2}\partial ( \lambda^{c}\mu )} \biggr)_{p,n_{1}} = -\frac{1}{T}C_{p,2}\bigl(\lambda^{a}\varepsilon,\lambda^{b}p,\lambda^{c}\mu\bigr)$$

with an expression *C*_*p*,2_(*λ* ^*a*^ *ε*,*λ* ^*b*^ *p*,*λ* ^*c*^ *μ*) which becomes constant at constant temperature and pressure:

16 $$ C_{p,2} = \mu^{ - \tfrac{2a + c - 1}{c}}C_{p,2}^{ \circ}$$

17 $$ C_{p,2} = C_{p,2}^{ \circ}\mu^{ - \alpha'}$$

and the critical index *α*′:

18 $$ \alpha' = \frac{2a + c - 1}{c}$$

Relations for other thermodynamic functions can also be derived.

For the partial molar isothermal compressibility we obtain:

19 $$ \kappa_{T,2} = \kappa_{T,2}^{ \circ}\mu^{\gamma'}$$

20 $$ \gamma' = \frac{2b + c - 1}{c}$$

Analogously, the relation for the pressure dependence of the partial molar volumes in terms of the scaling law has the form:

21 $$ V_{2} = V_{2}^{ \circ} p^{\tfrac{1}{\delta'}}$$

22 $$ \delta' = \frac{b}{1 - b - c}$$

Similar to the original theory [1] where the relation *ξ*∝*μ* ^−*ν*^ can be proven, we suspect the relation for the “partial molar correlation length”, a property which characterizes only the solute, is in the form:

23 $$ \xi_{2} = \xi_{2}^{ \circ}\mu^{ - \nu'}$$

with the critical index *ν*′.

In the conventional theory some relations between the critical indices, e.g. Rushbrooke relation *α*+2*β*+*γ*=2 or Widom relation *β*(*δ*−1)=*γ*, can be proved [1]. The modified theory causes some of these relations to have different forms:

24 $$\alpha' + 2\beta' + \gamma' =\frac{2a + c - 1 + 2 - 2b - 2c + 2b +c - 1}{c} = \frac{2a}{c} \ne2 $$

but they can be transformed into the original form for the case when the temperature, rather than molality, is a variable and consequently the constant *a* is the denominator of the fraction of Eq. 24:

25 $$ c \to a \quad \Rightarrow\quad \frac{2a}{c} = 2$$

Some other relations remain unchanged:

26 $$ \beta'\bigl(\delta' - 1\bigr) =\frac{1 - b - c}{c} \biggl( \frac{b}{1 - b - c} - 1 \biggr) = \frac {2b + c - 1}{c} =\gamma'$$

In the conventional approach the so called geometrical relation can be assumed to be in the form:

27 $$ \frac{G}{kTV} \propto\xi^{ - d}$$

resulting from the fact that thermodynamic potential divided by *kTV* has the same dimension as the inverse of volume, which can be expressed, using the correlation length, as *ξ* ^−*d*^.

Taking into account that heat capacity is the second derivative of the thermodynamic potential, the behavior of the Gibbs energy can be described using the heat capacity critical index *α* in the form of *G*∝*ε* ^2−*α*^. Correlation length, in turn, in terms of scaling can be written as *ξ*∝*ε* ^−*ν*^. Comparison of the indices of both functions results in the relation which links the critical index *α* (describing the behavior of heat capacity) with the dimensionality of the system *d*, by means of the critical index *ν* which describes the behavior of the correlation length [7], namely:

28 $$ 2 - \alpha= \nu d$$

The modification of the theory makes that we cannot be sure if this relation is true in the case of a solution. However, assuming the relation:

29 $$ 2 - \alpha' = \nu'd'$$

by analogy to Eq. 28, we can see that *d*′ denotes the dimensionality with respect to solutions, by analogy with the conventional approach, but a precise definition at this stage is difficult, due the lack of appropriate experimental data.

Agreement of the relations between the critical indices indicates the possibility that our treatment of the scaling theory could be applied to the description of solutions and phase transitions induced by changes of solution composition.

The conventional scaling theory can be used for the description of the continuous phase transitions (second order), in the case where the order parameter changes continuously. It can be extended, however, to the case of noncontinuous phase transitions (first order). Such an extension is described by the two-point scaling theory [2, 3], which is considered when the system can appear in *n* phases confined by the stability points of each phase. The two-point scaling is also based on the assumption that the thermodynamic potentials are generalized homogeneous functions, although their singularities are related to the stability points of each phase instead of the phase transition points which now are situated in the intervals between stability points. This approach results in a different temperature scale for each phase. The values of *m*_*sp*_ denote the stability points for lower limits of the concentration of phase *s* while the values *m*_*sf*_ represent the stability points for upper limits of the concentration of the phase *s*. In our case it refers to the regular solution of surfactant monomers (*s*=1) and the phase of micellar structures (*s*=2), respectively. In the case of a regular solution of surfactant monomers we consider only the upper stability point, and therefore we denote it simply as *m*_*f*_. Similarly for the micellar phase we consider only the lower limit denoted as *m*_*p*_.

The crossing points of the curves for each phase indicate the critical molality *m*_*c*_, which can be considered as the boundary between the concentration range in which the solution predominantly exhibits properties characteristic for a regular solution (*m*<*m*_*c*_) and the range in which the solution properties become typical for micellar systems (*m*>*m*_*c*_). This point corresponds to the transition point in the conventional approach to scaling.

Considering the two-point version of scaling theory, the relations for the heat capacities for each phase with respect to the molality variable *m* can be written as follows:

30a $$ C_{p,2}(m \le m_{f}) = C_{p,2}^{ \circ\, 1}\biggl( 1 - \frac{m}{m_{f}} \biggr)^{ - \alpha'_{1}}$$

30b $$ C_{p,2}(m \ge m_{p}) = C_{p,2}^{ \circ\, 2}\biggl( \frac{m}{m_{p}} - 1 \biggr)^{ - \alpha'_{2}}$$

Taking into account that the apparent molar quantities can be determined directly from experiment, the relations describing the concentration dependence of the apparent molar heat capacities can be derived in terms of scaling:

31a $$ C_{p,\Phi,2}(m \le m_{f}) = C_{p,2}^{ \circ\, 1}\biggl(\frac{1}{1 - \alpha'_{1}}\biggr)\frac{m_{f}}{m} \biggl[ q_{f}^{ \circ} -\biggl( 1 - \frac{m}{m_{f}} \biggr)^{1 - \alpha'_{1}} \biggr]$$

31b $$ C_{p,\Phi,2}(m \ge m_{p}) = C_{p,2}^{ \circ\, 2}\biggl(\frac{1}{1 - \alpha'_{2}}\biggr)\frac{m_{p}}{m} \biggl[ q_{p}^{ \circ} +\biggl( \frac{m}{m_{p}} - 1 \biggr)^{1 - \alpha'_{2}} \biggr]$$

For the calculation of the partial molar heat capacities *C*_*p*,2_ the derivative of the apparent molar heat capacity *C*_*p*,Φ,2_ is needed:

32 $$ C_{p,2} = C_{p,\Phi,2} + m\frac{\mathrm{d}C_{p,\Phi ,2}}{\mathrm{d}m}$$

and this quantity can be also expressed in terms of scaling: 33a
33b


Similar equations can be written for each phase in the case of partial molar volumes:

34a $$ V_{2}(m \le m_{f}) = V_{2}^{ \circ\, 1}\biggl( 1 - \frac{m}{m_{f}} \biggr)^{\beta'_{1}}$$

34b $$ V_{2}(m \ge m_{p}) = V_{2}^{ \circ\,2}\biggl( \frac{m}{m_{p}} - 1 \biggr)^{\beta'_{2}}$$

as well as for the apparent molar volumes:

35a $$ V_{\Phi,2}(m \le m_{f}) = V_{2}^{ \circ\, 1}\biggl(\frac{1}{1 + \beta'_{1}}\biggr)\frac{m_{f}}{m} \biggl[ q_{f}^{ \circ} -\biggl( 1 - \frac{m}{m_{f}} \biggr)^{1 + \beta'_{1}} \biggr]$$

35b $$ V_{\Phi,2}(m \ge m_{p}) = V_{2}^{ \circ\, 2} \biggl(\frac {1}{1 +\beta'_{2}}\biggr)\frac{m_{p}}{m} \biggl[ q_{p}^{ \circ} + \biggl(\frac{m}{m_{p}} - 1 \biggr)^{1 + \beta'_{2}} \biggr]$$

and their derivatives: 36a
36b


**Fig. 1:**
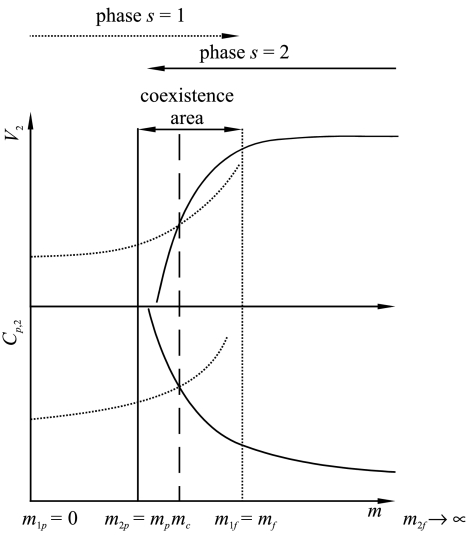
Qualitative influence of molality on the partial molar heat capacity and on the partial molar volume according to the two-point scaling theory

The shape of the curve for the partial molar heat capacity (and for the partial molar volume) versus molality *following* from the two-point scaling theory is the same as the shape of the curve for the heat capacity (and magnetization, respectively) versus temperature following from the conventional scaling theory. The graphical representation of the obtained results is showed in Fig. 1.

In the original theory the magnetization denotes the order parameter, the value of which is equal to zero after the transition point, and differs from zero before the critical point. In our case the partial molar volumes can also correspond to an order parameter whose behavior is characterized as follows: the parameter has a more or less constant value before the *c.m.c.* while it starts to increase after the *c.m.c.*

To determine if our approach is applicable and if the phase transition in a solution obeys the scaling laws, comparison with experimental data is necessary. We decided to study representative compounds of a few different types of surfactants and test our approach.

In our previous research we investigated solutions of the cationic surfactant decyltrimethylammonium bromide (C_10_TAB) [4], and the microheterogeneous aqueous solutions of 2-butoxyethanol [5] and 2-(2-hexyloxyethoxy)ethanol [6].

Equations 30a, 30b to 33a, 33b, which describe the concentration dependence of heat capacity, obtained as the result of the theory modification, are the same as those used previously [5, 6], Thus, we can see that our “intuitive” approach was correct, and the results obtained can be discussed in terms of the modified theory, presented now in a more rigorous form.

In order to extend the group of systems on which we test our approach, in the present paper we study the heat capacity of the solutions of a representative of another group of the surfactants, the anionic surfactant sodium octanoate (OctNa).

## Experimental

According to De Lisi et al. [8] the alkyltrimethylammonium bromides undergo micellize in a different way than the carboxylates since their apparent molar heat capacity goes through a maximum at the *c.m.c.*, while for carboxylates a “hump” at *c.m.c.* can be observed. These authors have analyzed the influence of composition on the apparent molar heat capacity, the apparent molar relative enthalpies and the apparent molar volumes of sodium decanoate with a “phase-separation” model, and they attributed the “hump” at the *c.m.c.* to the “relaxation” contribution.

Sodium octanoate in aqueous solution has quite a high *c.m.c.* [9], which is crucial from the point of view of calorimetric experiments.

Sodium octanoate micelles have a small aggregation number [10–14]. It is known from the computer simulation [10–14] that, in the case of small micelles such as those of OctNa, large thermal fluctuations are present.

González-Pérez et al. investigated sodium octanoate aqueous solutions in the molality ranges 0.04–0.9 mol⋅kg^−1^ [9] and 0.4–2.0 mol⋅kg^−1^ [15]. From the speed of sound and density versus molality isotherms the authors calculated *c.m.c.* values which decrease from 0.3828 mol⋅kg^−1^ at 298.15 K to 0.3589 mol⋅kg^−1^ at 318.15 K [9]. The same authors observed also the point called the critical micelle transition (*c.m.t.*) [15] at which the transition from spherical to nonspherical micelles occurs. The obtained *c.m.t.* values have a maximum value of 0.95 mol⋅kg^−1^ at 308 K [15]. The values were calculated from the density and conductivity data but it should be pointed out that the changes in these functions were very small and the criteria for determination of the *c.m.t.* are doubtful. In the case of adiabatic compressibilities the authors did not observe a change of slope but only an inversion of the temperature behavior of compressibilities at the point corresponding to *c.m.t.* The authors calculated the apparent molar volumes and compressibilities of the surfactant in water but did not present the appropriate values or graphs.

Apparent molar volumes and adiabatic compressibilities at 298.15 K in the molality range up to 2 mol⋅kg^−1^ were investigated also by Huang et al. [16, 17], who observed that the apparent molar volume of surfactant increases slightly up to 0.4 mol⋅kg^−1^ after which it starts to increase rapidly. At higher concentrations it remains almost constant. Huang et al. [16, 17] determined aggregation numbers equal to 10–15 [17], but did not observe the *c.m.t.* A similar value, equal to 16 at 293.15 K was obtained by D. Adair et al. [18] from viscosity measurements.

Aqueous solutions of sodium octanoate were extensively investigated by Ekwall et al. [19–25]. By analyzing the influence of composition on partial molar volumes, the authors indicate the existence of three critical points of micellization. Rapid increases of apparent molar volumes were observed at 0.38–0.52 mol⋅dm^−3^, 1.15–1.41 mol⋅dm^−3^, and 2.4–3.1 mol⋅dm^−3^ intervals; however, the increase in the second region is much smaller than in the first and the change in the third region is not pronounced. On the basis of viscosity measurements, Ekwall et al. [20, 21] found that spherical micelles existed in the solution in the concentration range up to 1.9 mol⋅dm^−3^. Above this concentration cylindrical micelles were observed.

Hayter and Zemb [26] investigated OctNa aqueous solutions using small angle neutron scattering (SANS) in the concentration range between the *c.m.c.* and *c.m.t.* values suggested by other authors. They observed a linear increase of the aggregation number with increasing concentration. The aggregation number obtained changed from 15 at the concentration of 0.60 mol⋅dm^−3^ up to 23 for a 1.20 mol⋅dm^−3^ solution. An extrapolation gives the value 13±1 for a solution at the concentration equal to *c.m.c.* On the basis of the results obtained, the authors [26] suggest a continuous increase of the micelle size rather than a transition to spherical structure.

Apparent molar heat capacities and volumes were investigated by Rosenholm et al. [27, 28] who observed a maximum in the apparent molar heat capacity versus molality curve as well as a rapid increase of the apparent molar volumes in the concentration range above 0.37 mol⋅dm^−3^. These measurements were only at 298.15 K and not over a large concentration range, and this was also a reason for us to investigate this system.

### Experimental

Sodium octanoate (Sigma-Aldrich ≥98%) was used as received without further purification. Deionized water was triply distilled in an argon atmosphere and degassed under vacuum. All solutions were prepared by weight.

The heat capacities under constant pressure were measured by means of a high sensitivity differential calorimeter Micro DSC III (Setaram, France) based on the Calvet principle. The *c*_*p*_ measurements were carried out within the temperature range 285.15–358.15 K using the “continuous with reference” mode. In this method the differential heat flow, between a cell filled with the investigated liquid and a reference, occurring during a continuous increase of calorimeter temperature is determined. In the temperature range under investigation, the scanning rate was 0.35 K⋅min^−1^. For measurements we used a batch-type cell of about 1 cm^3^ volume. The *c*_*p*_ values for each temperature were calculated from *c*_*p*_=*f*(*T*) function, by interpolation. As a reference substance of known heat capacity, water was used. Using the procedure developed in our laboratory and described widely by Góralski et al. [29], the uncertainty in the *c*_*p*_ values can be estimated to be smaller than 0.25% with an error in the absolute temperature determination of 0.05 K.

The densities of all surfactant solutions were measured using a flow densimeter (Sodev, model 03, Sherbrook, Quebec). The densimeter was calibrated with reference to pure water and nitrogen gas (absolute 1 atm). The density of water was taken from Kell [30] and that of nitrogen was calculated from the van der Waals equation of state. The reproducibility of the density measurements was 5×10^−6^ g⋅cm^−3^ and the uncertainty was estimated as 2×10^−5^ g⋅cm^−3^. The stability of the temperature in the densimeter (±0.001 K) was achieved with a closed loop thermostat and controlled using a special device calibrated with a PT 100 thermometer that allowed estimation of the absolute uncertainty in the temperature as 0.02 K. The measurements were performed under static conditions.

All solutions were prepared by weight with a mean uncertainty in the molality of 2×10^−5^ mol⋅kg^−1^.

### Results

#### Heat Capacity

From the calorimetrically determined *c*_*p*_ (listed in Table S1 of supplementary material) data the apparent molar heat capacities were calculated from the following equation:

37 $$ C_{p,\Phi,2} = M_{2}c_{p} +\frac{1000(c_{p} - c_{p,1}^{*})}{m_{2}}$$

**Fig. 2:**
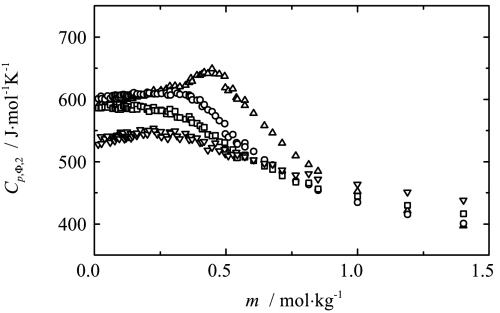
Influence of composition on the apparent molar heat capacity of sodium octanoate in aqueous solutions. Symbols represent experimental results at △ 293.15 K, ○ 313.15 K, □ 333.15 K and ▽ 353.15 K

The partial molar heat capacities were then calculated according to Eq. 32. This procedure was previously used for the determination of partial molar heat capacities of other colloidal systems and satisfactory agreement was found with data available in the literature [4, 5]. The values of the apparent molar heat capacities of sodium octanoate in aqueous solution, obtained as a function of molality at four temperatures, are shown in Fig. 2. For each temperature a maximum can be observed at a salt molality of about 0.3–0.5 mol⋅kg^−1^, which shifts to the higher values with increasing temperature. At the lowest temperature the maximum is most pronounced. The locations of these maxima correlate qualitatively with the *c.m.c.* values available in the literature [9, 15]. The shape of the curve for the partial molar heat capacity versus molality, and its values, correspond well with those investigated by Rosenholm et al. [27, 28]. At low molality, the apparent molar heat capacities increase with increasing temperature. In the salt molality range 0.6–1.0 mol⋅kg^−1^ this relation starts to change and, at high molality, inverse behavior of *C*_*p*,Φ,2_ is observed. Within the same composition range González-Pérez et al. observed similar behavior for the adiabatic compressibilities and, on the basis of conductometry, the authors [15] also identified the *c.m.t.* within that range.

**Fig. 3:**
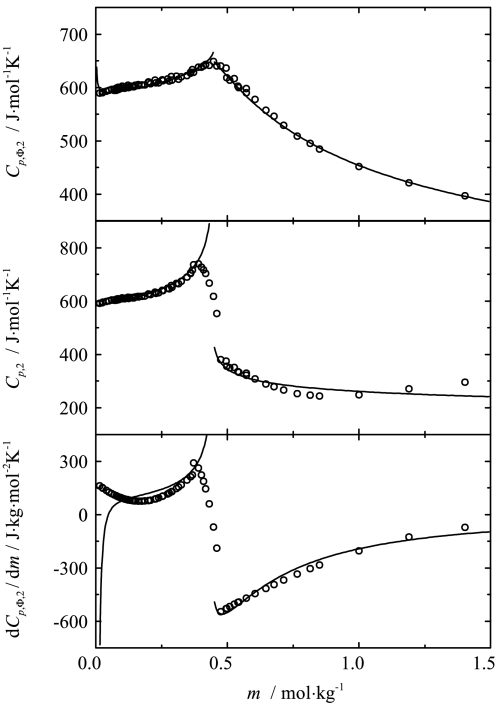
Influence of composition on the apparent, partial and the derivative of apparent molar heat capacities of sodium octanoate in aqueous solution at 293.15 K. Curves represent the best fit of the two-point scaling equations

**Fig. 4:**
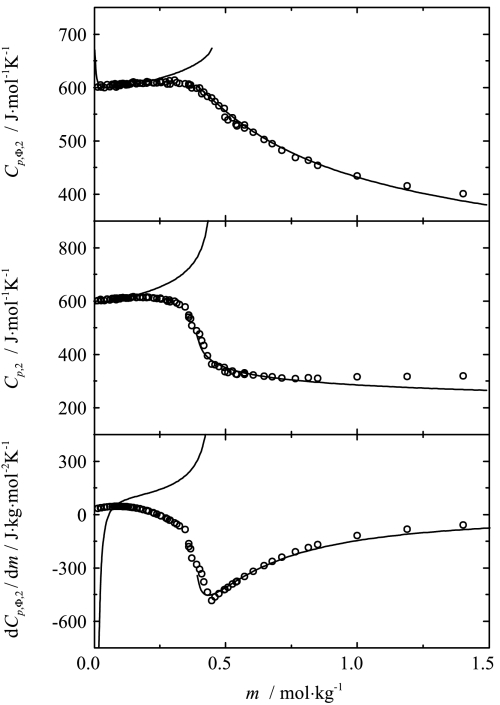
Influence of composition on the apparent, partial and the derivative of apparent molar heat capacities of sodium octanoate in aqueous solution at 313.15 K. Curves represent the best fit of the two-point scaling equations

**Fig. 5:**
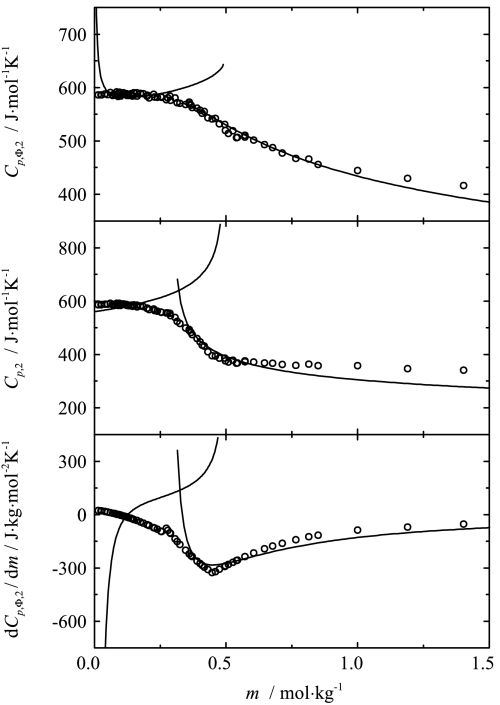
Influence of composition on the apparent, partial and the derivative of apparent molar heat capacities of sodium octanoate in aqueous solution at 333.15 K. Curves represent the best fit of the two-point scaling equations

**Fig. 6:**
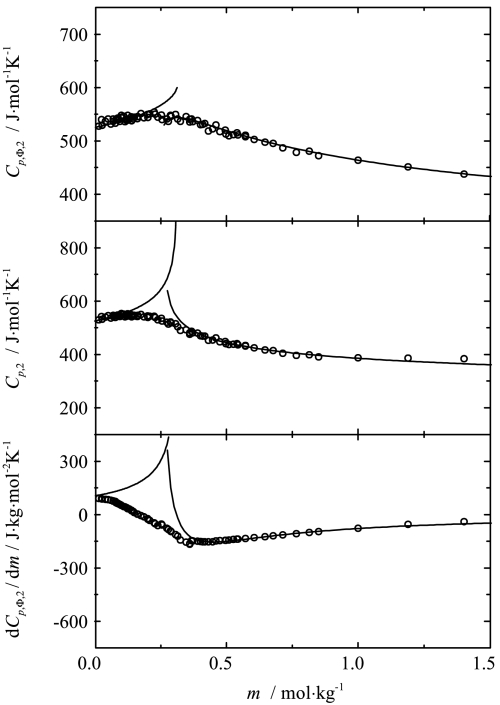
Influence of composition on of the apparent, partial and the derivative of apparent molar heat capacities of sodium octanoate in aqueous solution at 353.15 K. Curves represent the best fit of the two-point scaling equations

To analyze the data, the two-point scaling approach was applied. The apparent and partial molar heat capacities as well as the derivatives of the apparent molar heat capacities were fitted simultaneously to Eqs. 31a, 31b, 30a, 30b and 33a, 33b, respectively, for both phases. The results of this fitting for the four chosen temperatures are shown in Figs. 3, 4, 5, 6 together with the experimental points. The values of the critical indices obtained and other fitting parameters are given in Tables 1 and 2.

**Table 1:** The values of the critical exponents $\alpha'_{1}$, $\alpha'_{2}$, stability points *m*_*f*_, *m*_*p*_, constants $C_{p,2}^{ \circ\, 1},C_{p,2}^{ \circ\, 2}$, and the critical molality *m*_*c*_ of the considered system

*T*/K	$\alpha'_{1}$	$\alpha'_{2}$	*m*_*f*_/mol⋅kg^−1^	*m*_*p*_/mol⋅kg^−1^	$C_{p,2}^{ \circ\, 1}\allowbreak/\mbox {J}{\cdot}\mbox{mol}^{-1}{\cdot}\mbox{K}^{-1}$	$C_{p,2}^{ \circ\, 2}\allowbreak/ \mbox {J}{\cdot}\mbox{mol}^{-1}{\cdot}\mbox{K}^{-1}$	*m*_*c*_/mol⋅kg^−1^
293.15	0.125	0.125	0.446	0.440	582	269	0.4400
313.15	0.125	0.125	0.448	0.381	589	303	0.3813
333.15	0.125	0.125	0.490	0.338	560	357	0.3408
353.15	0.125	0.125	0.312	0.263	525	438	0.2711

**Table 2:** Values of the two fitting parameters $q_{f}^{ \circ}$ and $q_{p}^{ \circ}$ used for the description of the experimental behavior of the *C*_*p*,Φ,2_ and (d*C*_*p*,Φ,2_/d*m*) functions

*T*/K	$q_{f}^{ \circ}$	$q_{p}^{ \circ}$
293.15	1.0007	2.1197
313.15	1.0010	1.7454
333.15	1.0046	1.3920
353.15	1.0000	1.0586

#### Molar Volumes

From the measured density data (listed in Table S2 of supplementary material) the apparent molar volumes at 293.15 K were calculated from the following equation:

38 $$ V_{\Phi,2} = \frac{M_{2}}{d} + \frac{1000(d -d_{1}^{*})}{m_{2}dd_{1}^{*}}$$

where $d_{1}^{*}$ is density of the pure solvent (water). The partial molar volumes were calculated as follows:

39 $$ V_{2} = V_{\Phi,2} + m_{2}\frac{\mathrm{d}V_{\Phi,2}}{\mathrm{d}m_{2}}$$

**Fig. 7:**
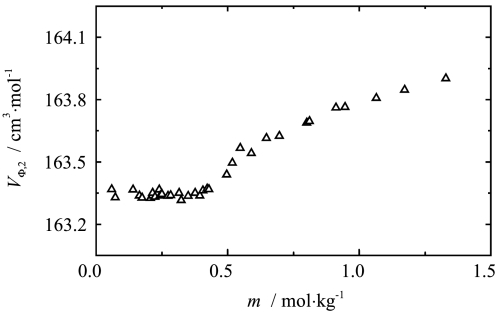
Influence of composition on the apparent molar volumes of sodium octanoate in aqueous solution at 293.15 K

The values of the apparent molar volumes of sodium octanoate in aqueous solution, as a function of molality at 293.15 K, are shown in Fig. 7.

**Fig. 8:**
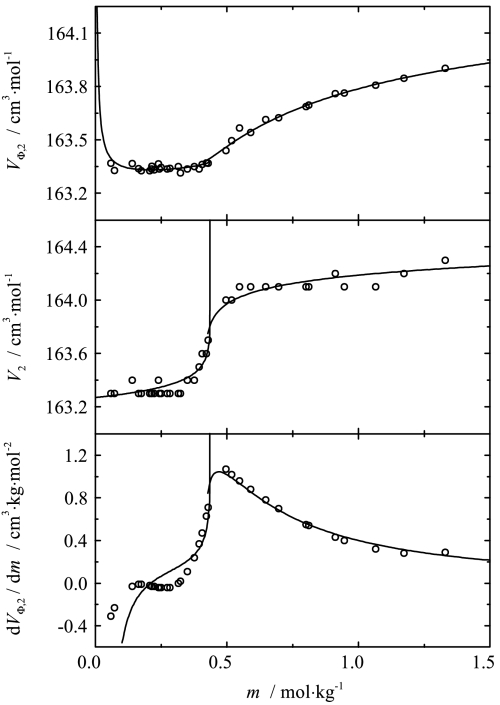
Influence of composition on the apparent, partial and the derivative of apparent molar volumes of sodium octanoate in aqueous solution at 293.15 K. Symbols represent experimental results. Curves represent the best fit of the two-point scaling equations

The two-point scaling approach was applied to the analysis of the data. The apparent, partial molar volumes and the derivatives of apparent molar volumes were fitted simultaneously to Eqs. 35a, 35b, 34a, 34b and 36a, 36b, respectively, for both phases. The results are shown in Fig. 8 together with the experimental data. The values of the critical indices obtained and other fitting parameters are given in Tables 3 and 4.

**Table 3:** Values of the critical exponents $\beta'_{1}$, $\beta'_{2}$, the stability points *m*_*f*_,*m*_*p*_, constants $V_{2}^{01}$, $V_{2}^{02}$, and the critical molality *m*_*c*_ of the considered system

*T*/K	$\beta'_{1}\allowbreak\times10^{3}$	$\beta'_{2}\allowbreak\times10^{3}$	*m*_*f*_/mol⋅kg^−1^	*m*_*p*_/mol⋅kg^−1^	$V_{2}^{ \circ\, 1}\allowbreak/ \mbox {cm}^{3}{\cdot}\mbox{mol}^{-1}$	$V_{2}^{ \circ\, 2}\allowbreak/\mbox {cm}^{3}{\cdot}\mbox{mol}^{-1}$	*m*_*c*_/mol⋅kg^−1^
293.15	−0.57	0.68	0.438	0.416	162	164	0.4180

**Table 4:** Values of the two fitting parameters $q_{o}^{f}$ and $q_{o}^{p}$ used for the description of the experimental behavior of the *V*_Φ,2_ and d*V*_Φ,2_/d*m* functions

*T*/K	$q_{f}^{ \circ }$	$q_{p}^{ \circ}$
293.15	1.0001	0.9960

## Discussion

As one can see from Figs. 3, 4, 5, 6 and 8, the influence of composition on the partial molar heat capacities as well as the partial molar volumes is subject to the scaling laws. The values of the critical indices obtained are temperature independent as was observed previously for all of the examined systems [4–6]. The parameter $\alpha'_{1}$ has also the same value $\alpha'_{1} = 0.125$ as in all the previously examined systems and the parameter $\alpha'_{2}$ has the same value as the parameter $\alpha'_{1}$. The same situation was observed previously for aqueous solutions of decyltrimethylammonium bromide. The values of critical molalities and their changes with the temperature are close to the values determined by means of different techniques given in literature [9, 15–17, 19–25]. Also the values of stability limits for each phase determined from the partial molar heat capacity behavior and the partial molar volume behavior are close to each other. Similar observations in the case of the critical molality additionally confirm that the micellization process is subject to the scaling laws.

As mentioned above, the critical index describing the behavior of the heat capacity in the conventional theory can be related to the dimensionality of the system by means of Eq. 28. For a precise calculation of the dimensionality, values of the $\nu'_{i}$ indices are necessary, but it can be observed that the values of $\alpha'_{i}$ are related to the structure of the solution. For the solutions of monomers (concentration range below *c.m.c.*), the values of $\alpha'_{1}$ obtained are equal to 0.125 for each previously analyzed system [4–6] as well as for the present system. For the micellar solutions, in which spherical micelles are formed (C_10_TAB [4], OctNa, present work), the $\alpha'_{2}$ index also takes the value 0.125. For microheterogeneous solutions (2-butoxyethanol [5], and 2-(2-hexyloxyethoxy)etanol aqueous solutions [6]), the value of $\alpha'_{2}$ is equal to 0.33 for both systems.

## Conclusion

The two-point scaling theory was modified in order to describe the phase transition in solution. Based on assumptions similar to those in the case of the conventional scaling theory and using the same procedures, relations describing the influence of composition on the partial molar heat capacities and volumes were derived.

The form of the relations obtained is the same as in our “intuitive” approach [5, 6], and the previously obtained results can be discussed in terms of the modified theory in the present paper.

Our approach can be extended to the discussion of some other thermodynamic properties such as the partial molar isothermal compressibilities. The appropriate equations can be derived, but the lack of experimental data prevents our obtaining the other critical indices and further discussion.

The values of the critical indices $\alpha'_{1}$ for the sodium octanoate monomer solution are the same as for all the previously investigated systems, and the values of $\alpha'_{2}$ are the same as for the decyltrimethylammonium bromide solutions, which could indicate universality of the micellization process. The value of the critical index $\alpha'_{2}$ depends on the structure of the aggregates formed. The stability points for each phase, and critical molalities determined from the scaling analysis of data from two independent measurements, were close to each other. The results obtained confirm that the aggregation process is subject to scaling laws and the scaling analysis can provide much useful information. Our model, based on scaling laws, seems to be more versatile than e.g. the phase separation model used by De Lisi et al. [8] for the analysis of the micellization of the carboxylates, which, as the authors suggest, does not work well for alkyltrimethylammonium bromides solutions. In our case we cannot distinguish the “relaxation” contribution. It seems to be incorporated in our model, as the scaling laws consider the singular part of the thermodynamic potentials. This “relaxation” effect is related to the shift of equilibrium when the temperature is changed [31]. This equilibrium means that the change of thermodynamic properties at *c.m.c.* can be “slow” (occurs over some concentration range). In our approach this fact is taken into account by the “phase coexistence” region, whose width (span) changes with the temperature, and depends on the type of surfactant. From this point of view our approach exhibits some advantages for analyzing the behavior of different types of surfactants.

## Electronic Supplementary Material

Below is the link to the electronic supplementary material.

Supplementary Tables for: Modification of the Two-Point Scaling Theory for the Description of the Phase Transition in Solution. Analysis of the Sodium Octanoate Aqueous Solutions (DOC 102 kB)
