# Supplementary material for: Modification of the Two-Point Scaling Theory for the Description of the Phase Transition in Solution. Analysis of Sodium Octanoate Aqueous Solutions
Source: J Solution Chem. 2012 Feb 2;41(2):318–34. doi: 10.1007/s10953-012-9795-6 (PMC3298654; doi:10.1007/s10953-012-9795-6)
Supplement: Supplementary file 1 — Supplementary Tables for: Modification of the Two-Point Scaling Theory for the Description of the Phase Transition in Solution. Analysis of the Sodium Octanoate Aqueous Solutions (DOC 102 kB) [file 10953_2012_9795_MOESM1_ESM.doc]

**Supplementary Tables for:**

**Modification of the Two-Point Scaling Theory for the Description of the Phase Transition in Solution. Analysis of the Sodium Octanoate Aqueous Solutions**

Henryk Piekarski ∙ Michał Wasiak ∙ Leszek Wojtczak

**Table S1** Isobaric heat capacities at of OctNa solutions 293.15, 313.15, 333,15 and 353.15 K

| *m*  / mol∙kg^−1^ | *C_p_* / J∙g^−1^∙K^−1^ | | | |
| --- | --- | --- | --- | --- |
|  | 293.15 K | 313.15 K | 333.15 K | 353.15 K |
| 0.00000 | 4.1868 | 4.1819 | 4.1877 | 4.1998 |
| 0.01506 | 4.1852 | 4.1805 | 4.1861 | 4.1972 |
| 0.02498 | 4.1842 | 4.1796 | 4.1850 | 4.1958 |
| 0.02753 | 4.1840 | 4.1793 | 4.1847 | 4.1951 |
| 0.04010 | 4.1827 | 4.1781 | 4.1834 | 4.1933 |
| 0.05016 | 4.1818 | 4.1774 | 4.1823 | 4.1920 |
| 0.06046 | 4.1809 | 4.1763 | 4.1814 | 4.1898 |
| 0.07005 | 4.1798 | 4.1756 | 4.1801 | 4.1886 |
| 0.07012 | 4.1800 | 4.1757 | 4.1800 | 4.1889 |
| 0.07774 | 4.1791 | 4.1751 | 4.1794 | 4.1871 |
| 0.07966 | 4.1791 | 4.1749 | 4.1791 | 4.1873 |
| 0.07987 | 4.1792 | 4.1749 | 4.1790 | 4.1873 |
| 0.08498 | 4.1788 | 4.1740 | 4.1790 | 4.1865 |
| 0.08715 | 4.1783 | 4.1742 | 4.1784 | 4.1864 |
| 0.09033 | 4.1782 | 4.1737 | 4.1777 | 4.1857 |
| 0.09444 | 4.1779 | 4.1735 | 4.1780 | 4.1848 |
| 0.09927 | 4.1774 | 4.1729 | 4.1767 | 4.1847 |
| 0.10045 | 4.1776 | 4.1732 | 4.1769 | 4.1849 |
| 0.11025 | 4.1763 | 4.1724 | 4.1763 | 4.1833 |
| 0.11483 | 4.1762 | 4.1720 | 4.1757 | 4.1815 |
| 0.12089 | 4.1755 | 4.1713 | 4.1748 | 4.1808 |
| 0.12247 | 4.1759 | 4.1710 | 4.1748 | 4.1809 |
| 0.12528 | 4.1750 | 4.1710 | 4.1741 | 4.1814 |
| 0.13091 | 4.1748 | 4.1704 | 4.1735 | 4.1796 |
| 0.13840 | 4.1742 | 4.1699 | 4.1728 | 4.1790 |
| 0.13943 | 4.1739 | 4.1700 | 4.1730 | 4.1779 |
| 0.14487 | 4.1734 | 4.1697 | 4.1718 | 4.1775 |
| 0.15023 | 4.1734 | 4.1695 | 4.1722 | 4.1772 |
| 0.16126 | 4.1726 | 4.1681 | 4.1711 | 4.1752 |
| 0.16216 | 4.1722 | 4.1682 | 4.1700 | 4.1757 |
| 0.17514 | 4.1713 | 4.1671 | 4.1695 | 4.1748 |
| 0.18695 | 4.1703 | 4.1660 | 4.1682 | 4.1717 |
| 0.19967 | 4.1703 | 4.1651 | 4.1660 | 4.1710 |
| 0.20120 | 4.1700 | 4.1655 | 4.1662 | 4.1698 |
| 0.20843 | 4.1690 | 4.1647 | 4.1644 | 4.1700 |
| 0.22551 | 4.1691 | 4.1632 | 4.1640 | 4.1684 |
| 0.22893 | 4.1676 | 4.1629 | 4.1626 | 4.1671 |
| 0.23902 | 4.1669 | 4.1621 | 4.1615 | 4.1644 |
| 0.25337 | 4.1670 | 4.1618 | 4.1602 | 4.1634 |
| 0.25552 | 4.1665 | 4.1608 | 4.1597 | 4.1610 |
| 0.27338 | 4.1660 | 4.1597 | 4.1566 | 4.1587 |
| 0.27947 | 4.1645 | 4.1583 | 4.1569 | 4.1567 |

*Table continues on next page*

**Table S1** (*continued*)

| *m*  / mol∙kg^−1^ | | *C_p_* / J∙mol^−1^∙K^−1^ | | | | | | |
| --- | --- | --- | --- | --- | --- | --- | --- | --- |
|  |  | 293.15 K | | 313.15 K | | 333.15 K | | 353.15 K |
| 0.28744 | 4.1662 | | 4.1592 | | 4.1570 | | 4.1586 | |
| 0.28912 | | 4.1642 | | 4.1575 | | 4.1547 | | 4.1578 |
| 0.30636 | | 4.1651 | | 4.1582 | | 4.1541 | | 4.1565 |
| 0.31517 | | 4.1629 | | 4.1563 | | 4.1504 | | 4.1528 |
| 0.32542 | | 4.1634 | | 4.1549 | | 4.1489 | | 4.1499 |
| 0.34630 | | 4.1628 | | 4.1531 | | 4.1459 | | 4.1497 |
| 0.35991 | | 4.1641 | | 4.1519 | | 4.1447 | | 4.1462 |
| 0.36038 | | 4.1634 | | 4.1510 | | 4.1458 | | 4.1444 |
| 0.36692 | | 4.1653 | | 4.1507 | | 4.1432 | | 4.1454 |
| 0.37130 | | 4.1633 | | 4.1484 | | 4.1409 | | 4.1430 |
| 0.38964 | | 4.1657 | | 4.1467 | | 4.1384 | | 4.1413 |
| 0.40174 | | 4.1651 | | 4.1458 | | 4.1356 | | 4.1366 |
| 0.40985 | | 4.1656 | | 4.1411 | | 4.1325 | | 4.1356 |
| 0.41804 | | 4.1663 | | 4.1414 | | 4.1326 | | 4.1351 |
| 0.43116 | | 4.1649 | | 4.1369 | | 4.1263 | | 4.1275 |
| 0.44767 | | 4.1674 | | 4.1341 | | 4.1231 | | 4.1267 |
| 0.46012 | | 4.1632 | | 4.1300 | | 4.1219 | | 4.1279 |
| 0.47531 | | 4.1623 | | 4.1249 | | 4.1155 | | 4.1201 |
| 0.49524 | | 4.1597 | | 4.1204 | | 4.1121 | | 4.1183 |
| 0.49808 | | 4.1511 | | 4.1127 | | 4.1066 | | 4.1144 |
| 0.50956 | | 4.1483 | | 4.1088 | | 4.1022 | | 4.1110 |
| 0.52658 | | 4.1485 | | 4.1084 | | 4.1021 | | 4.1095 |
| 0.53979 | | 4.1406 | | 4.1004 | | 4.0936 | | 4.1090 |
| 0.54342 | | 4.1391 | | 4.0987 | | 4.0932 | | 4.1067 |
| 0.57229 | | 4.1358 | | 4.0956 | | 4.0909 | | 4.1017 |
| 0.57252 | | 4.1315 | | 4.0923 | | 4.0891 | | 4.1004 |
| 0.60552 | | 4.1217 | | 4.0835 | | 4.0807 | | 4.0924 |
| 0.64628 | | 4.1061 | | 4.0697 | | 4.0691 | | 4.0826 |
| 0.67704 | | 4.0956 | | 4.0601 | | 4.0609 | | 4.0765 |
| 0.71422 | | 4.0805 | | 4.0462 | | 4.0480 | | 4.0648 |
| 0.76576 | | 4.0599 | | 4.0283 | | 4.0321 | | 4.0506 |
| 0.81418 | | 4.0430 | | 4.0157 | | 4.0226 | | 4.0439 |
| 0.85015 | | 4.0293 | | 4.0024 | | 4.0088 | | 4.0315 |
| 1.00007 | | 3.9776 | | 3.9585 | | 3.9717 | | 3.9988 |
| 1.19016 | | 3.9139 | | 3.9041 | | 3.9231 | | 3.9543 |
| 1.40321 | | 3.8465 | | 3.8471 | | 3.8696 | | 3.9038 |
| 1.73225 | | 3.7611 | | 3.7726 | | 3.8004 | | 3.8362 |

**Table S2** Densities of OctNa solutions at 293.15 K.

| *m* | *d* |
| --- | --- |
| / mol∙kg^−1^ | / g∙cm^−3^ |
| 0.00000 | 0.99824 |
| 0.05913 | 0.99842 |
| 0.07267 | 0.99846 |
| 0.14108 | 0.99867 |
| 0.16484 | 0.99874 |
| 0.17490 | 0.99877 |
| 0.20690 | 0.99887 |
| 0.21347 | 0.99889 |
| 0.21501 | 0.99889 |
| 0.22444 | 0.99892 |
| 0.24015 | 0.99896 |
| 0.24383 | 0.99897 |
| 0.25063 | 0.99899 |
| 0.27298 | 0.99906 |
| 0.28415 | 0.99909 |
| 0.31553 | 0.99918 |
| 0.32345 | 0.99921 |
| 0.35058 | 0.99928 |
| 0.37593 | 0.99934 |
| 0.39420 | 0.99940 |
| 0.40574 | 0.99942 |
| 0.42227 | 0.99946 |
| 0.42895 | 0.99948 |
| 0.49644 | 0.99963 |
| 0.51835 | 0.99966 |
| 0.54821 | 0.99970 |
| 0.58983 | 0.99982 |
| 0.64722 | 0.99991 |
| 0.69722 | 1.00002 |
| 0.80081 | 1.00021 |
| 0.80149 | 1.00021 |
| 0.81150 | 1.00023 |
| 0.91190 | 1.00039 |
| 0.94674 | 1.00046 |
| 1.06541 | 1.00066 |
| 1.17256 | 1.00082 |
| 1.32992 | 1.00105 |
| 1.50218 | 1.00130 |
